# Distinct uric acid trajectories are associated with incident cardiac conduction block

**DOI:** 10.1186/s13075-024-03288-8

**Published:** 2024-02-27

**Authors:** Na Li, Liufu Cui, Rong Shu, Haicheng Song, Jierui Wang, Shuohua Chen, Gary Tse, Nan Zhang, Xuemei Yang, Wenqi Xu, Shouling Wu, Tong Liu

**Affiliations:** 1 Department of Cardiology, Tianjin Institute of Cardiology, Second Hospital of Tianjin Medical University, Tianjin Key Laboratory of Ionic-Molecular Function of Cardiovascular Disease, No. 23, Pingjiang Road, Hexi District, Tianjin, 300211 People’s Republic of China; 2 Department of Rheumatology and Immunology, Kailuan General Hospital, North China University of Science and Technology, Tangshan, China; 3 Department of Cardiology, Kailuan General Hospital, North China University of Science and Technology, Tangshan, China; 4 School of Nursing and Health Studies, Hong Kong Metropolitan University, Hong Kong, China; 5 School of Clinical Medicine, North China University of Science and Technology, Tangshan, Hebei China

**Keywords:** Uric acid, Trajectories, Cardiac conduction block, Risk factors

## Abstract

**Background:**

The association of longitudinal uric acid (UA) changes with cardiac conduction block risk is unclear. We aimed to identify the trajectories of UA and explore its association with cardiac conduction block.

**Methods:**

A total of 67,095 participants with a mean age of 53.12 years were included from the Kailuan cohort in Tangshan, China, who were free of cardiac conduction block and with repeated measurements of UA from 2006 to 2012. UA trajectories during 2006 to 2012 were identified by group-based trajectory modeling. Cox proportional hazard regression models were used to assess the association of UA trajectories with cardiac conduction block.

**Results:**

We categorized three observed discrete trajectories of UA during 2006–2012 period: low-stable, moderate-stable, and high-stable. Over a median follow-up of 6.19 years, we identified 1405 (2.09%) incident cardiac conduction block. Compared to those in the low-stable trajectory, the adjusted hazard ratios (HRs) (95% confidence interval [CI]) of cardiac conduction block in the moderate-stable and high-stable trajectory were 1.30 (1.16–1.47) and 1.86 (1.56–2.22), and HRs of atrioventricular block were 1.39 (1.12–1.72) and 2.90 (2.19–3.83), and HRs of bundle branch blocks were 1.27 (1.10–1.47) and 1.43 (1.13–1.79). Notably, although the average UA level in the moderate-stable UA trajectory group is within the normal range, the risk of cardiac conduction block has increased.

**Conclusions:**

The moderate-stable and high-stable trajectories are associated with increased risk for new-onset cardiac conduction block. Monitoring UA trajectories may assist in identifying subpopulations at higher risk for cardiac conduction block.

**Supplementary Information:**

The online version contains supplementary material available at 10.1186/s13075-024-03288-8.

## Introduction

Cardiac conduction block is a type of bradyarrhythmia, mainly caused by fibrosis of the conduction system and related to myocardial fibrosis [1, 2], while it can occur at any level of the cardiac conduction system. In the worst case, progression of this disorder can lead to heart failure and death [3]. There is currently limited data on the prevalence and risk factors of cardiac conduction block in large populations. However, accumulating evidence now suggests that even PR interval prolongation, first-degree atrioventricular block (AVB), or bundle branch blocks (BBB) are independently associated with a poor cardiac prognosis [4, 5]. As of today, the only available therapy for severe cardiac conduction block is the implantation of pacemakers [6]. Indeed, previous studies have shown that the risk factors for cardiac conduction block include hypertension, diabetes mellitus, electrolyte disorders, drugs, gene mutations, and ischemic heart disease [7–9]. However, there are currently no established prevention strategies for cardiac conduction block, so it is necessary to further identify the modifiable risk factors for cardiac conduction block.

Uric acid (UA) has been confirmed to play an essential role in the pathogenesis of cardiovascular disease (CVD) [10]. However, there is limited research on UA and cardiac conduction block. Mantovani et al*.* found that in patients with type 2 diabetes, compared with the first tertile, the risk of cardiac conduction defects in the third tertile of uric acid (UA) was nearly twofold increased [11]. Thus, UA may be a risk factor for cardiac conduction block. Nevertheless, this study focused only on the impact of a single UA value on cardiac conduction block, in a cross-sectional study, ignoring the effect of UA trajectories changing over time. The onset of cardiac conduction block is a slow process, and a single measurement of UA does not reflect the longitudinal variation associated with elevated UA levels. Previous studies have suggested that higher UA trajectories are associated with increased risks of CVD, diabetes, and metabolic syndrome [12–14]. The effect of long-term UA trajectories on cardiac conduction block incidence remains unknown.

As a consequence, the aims of the present study were to identify distinct UA trajectories over a 6-year exposure period and to explore the association between UA trajectories and cardiac conduction block risk based on data from the Kailuan study, a prospective population-based cohort.

## Materials and methods

### Study population

Data were obtained from the Kailuan study (trial registration number: ChiCTR-TNC-11001489), which is a community-based ongoing cohort study performed in Tangshan City, China. Study details have been described elsewhere [15]. Briefly, the Kailuan Study was designed and initiated in 2006–2007 and a total of 101,510 participants including 81,110 men and 20,400 women were enrolled to participate in the baseline surveys and the follow-up visits biennially. This study aimed to investigate the risk factors of cardiovascular diseases and other non-communicable diseases. The laboratory testing includes biochemical markers such as UA, as well as standard twelve lead electrocardiogram (ECG) data. The study was conducted according to the principles of the Declaration of Helsinki and was approved by the Ethics Committee of Kailuan Hospital. All participants provided written informed consent to their enrolment.

**Fig. 1:**
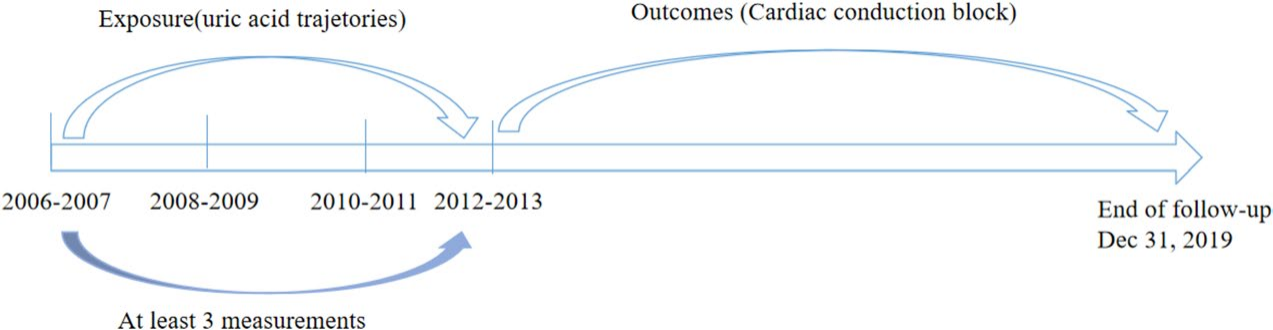
Time line of exposure and follow-up assessment of uric acid

In the present study, UA trajectories were identified according to the change in UA from 2006 to 2012 to predict the cardiac conduction block risk after 2012. The present study was restricted to the population who participated in at least 3 examinations from 2006 to 2012 (*n* = 87,669), with the last examination data during the exposure period as the baseline, the start time-point of follow-up (Fig. 1). Individuals were excluded if they had one of the following: (1) uric acid data of less than 3 times or without data of electrocardiogram at baseline (*n* = 2,939); (2) missing data of ECG during follow-up (*n* = 9,586); (3) treatment with beta-blocker or non-dihydropyridine calcium-channel blockers at baseline (*n* = 1,158); (4) a history of cardiac conduction block, myocardial infarction (MI), heart failure (HF), or atrial fibrillation (AF) at baseline (*n* = 6,891). Therefore, a total of 67,095 individuals were enrolled in the final analyses (Figure S1).

### Data collection and definitions

After an overnight fasting period (at least 8 h), fasting blood samples of 5 ml were taken from the anterior elbow vein in the morning, and the blood was transfused into vacuum tubes containing EDTA. All blood samples were measured by auto-analyzer (Hitachi 747; Hitachi, Tokyo, Japan). The laboratory tests included serum UA, high sensitivity C-reactive protein (hs-CRP), fasting blood glucose (FBG), triglycerides (TG), and blood creatinine. UA was detected by the oxidase method, with an intra- and interassay coefficient of variation ≤ 6%.

### ECG measurements and definition of endpoint events

Having the subject lying down in a quiet room for 5 min, a 10 s twelve-lead standard ECG was performed. The ECG interpretation was performed by experienced cardiologists. The diagnosis of cardiac conduction block was based on the ECG and confirmed by experienced cardiologists. In case of controversy, a senior cardiologist was consulted for a final diagnosis or until agreement was reached with discussion. The specific ECG abnormalities were classified according to Minnesota coded (MC) criteria [16], seeing Tables S1 for diagnostic criteria [6]. Cardiac conduction block was defined as any conduction disease, including AVB I–III degree, complete right bundle branch block (CRBBB), incomplete right bundle branch block (iRBBB), complete left bundle branch block (CLBBB), incomplete left bundle branch block (iLBBB), left anterior fascicular block (LAFB), left posterior fascicular block (LPFB), and non-specific intra-ventricular conduction delay (NS-IVCD).

### Assessment of the outcome

All the participants were followed from the baseline until the occurrence of cardiac conduction block, death, or the end of the follow-up (December 31, 2019), whichever came first. The subtypes of cardiac conduction block included AVB or BBB during the follow-up period. AVB included AVB I–III degree, while BBB included CRBBB, iRBBB, CLBBB, iLBBB, LAFB, and LPFB.

### Potential confounders

Demographic and clinical characteristics, including age, sex, smoking, drinking, physical exercise, and past self-described medical history (hypertension, diabetes, CVD, etc.) were collected for all Kailuan participants at every clinical follow-up visit. During the survey interview, trained staff assessed height, weight, and blood pressure. The body mass index (BMI) was calculated by body weight (kg) divided by height squared (m^2^). Overweight is defined as BMI ≥ 24 kg/m^2 ^[17]. Hypertension was defined as a self-reported history of hypertension, current treatment with an antihypertensive agent, or a measured systolic blood pressure (SBP) ≥ 140 mmHg, or diastolic blood pressure (DBP) ≥ 90 mmHg. Diabetes was defined as a self-reported history of diabetes, current treatment with a hypoglycemic agent, or FBG ≥ 7.0 mmol/L. Current alcohol consumption was defined as those who drank at least once a day in the past year, and current smoking was defined as smoking at least one cigarette a day on average during the past year. Physical exercise was defined as an exercise frequency ≥ 3 times/week and duration ≥ 30 min/time. The estimated glomerular filtration rate (eGFR) was calculated according to the formula of the Chronic Kidney Disease Epidemiology Cooperation (CKD-EPI) [18].

### Statistical analysis

**Fig. 2:**
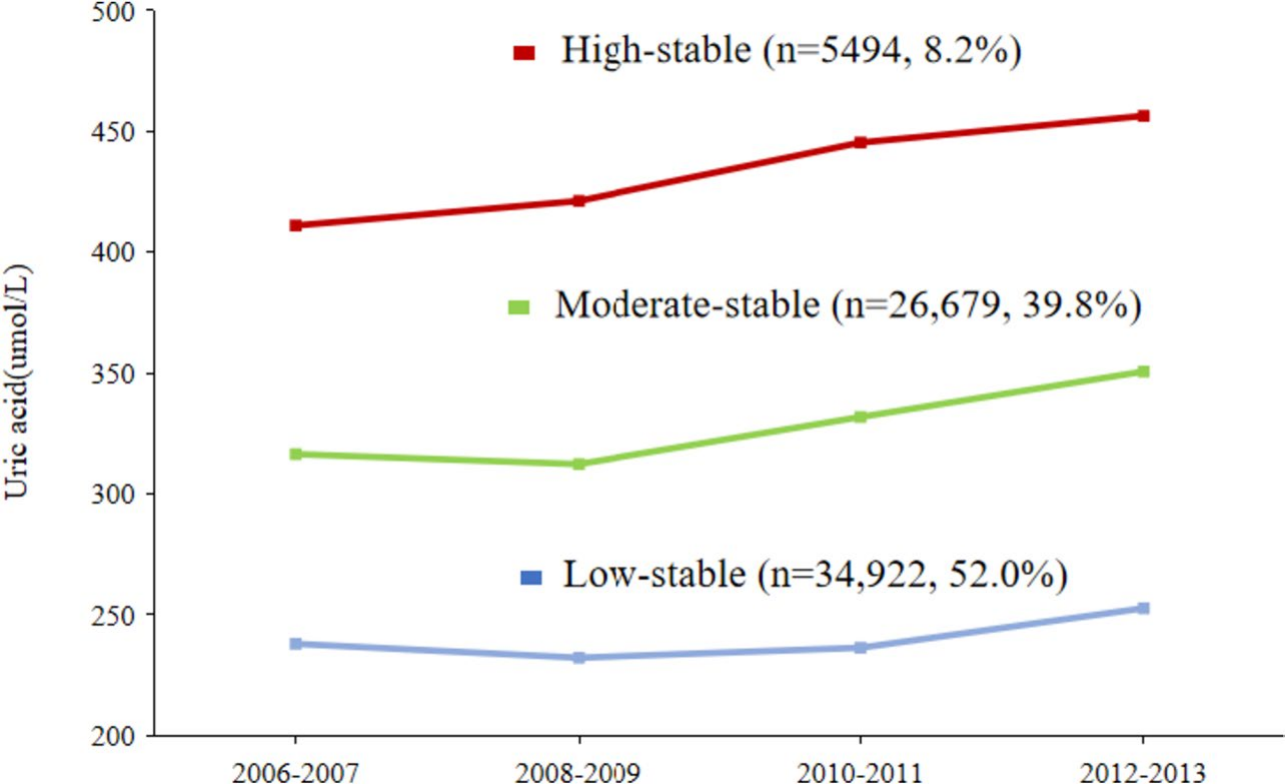
Dynamic trajectories of uric acid during the exposure period (2006–2012) in the study population

UA trajectories were identified by group-based trajectory modeling using SAS PROC TRAJ [19]. This method can automatically divide the study population into classes, in such a way that participants in the same class tend to have similar trajectories of UA change. The censored normal model was applied. First, model fit was assessed using the Bayesian information criterion (BIC), with the number of participants in each trajectory no less than 5% of the overall population. All UA trajectories started with quadratic shapes and compared the BIC with the models of two, three, four, and five classes. The results showed that the optimal number of trajectories was three. Second, we compared the model with different functional forms. Cubic, quadratic, and linear terms were considered and evaluated based on their significance level (*p* < 0.05), starting with the highest polynomial. In our final model, we had one pattern with quadratic order terms and two patterns with up to cubic order terms (Fig. 2).

Baseline characteristics were compared using ANOVA or the Kruskal–Wallis test for continuous variables according to their distribution, and the Chi-square test for categorical variables. The Kaplan–Meier method was performed to evaluate the incidence rate of cardiac conduction block while differences between groups were evaluated using the log-rank test. The incidence rate of cardiac conduction block per 1000 person-years was calculated. The proportional hazard assumptions were evaluated by visualization of Schoenfeld residuals, and no violation was observed. After confirming the satisfaction of the proportional risk hypothesis, multivariable Cox regression models were constructed to estimate the association between different trajectory groups and the risks of cardiac conduction block development by calculating the hazard ratios (HRs) and 95% confidence intervals (CIs). To adjust for potential confounding factors, 5 models were built systematically. Model 1 was adjusted for age and sex. Model 2 was further adjusted for smoking, drinking, physical activity, BMI, eGFR, hs-CRP, TG, hypertension, and diabetes. Model 3 was further adjusted for antihypertensive drugs, hypoglycemic drugs, and lipid-lowering drugs. Model 4 was adjusted for variables in Model 3 plus UA at the first visit. Model 5 was adjusted for variables in Model 3 plus UA at baseline. To explore whether UA trajectories exhibit different effects on the outcomes in special populations, subgroup analysis stratified by age (< 60 or ≥ 60 years), sex, BMI (< 24 or ≥ 24 kg/m^2^), and history of hypertension (no or yes) was performed; interaction between stratified variables and UA trajectories was tested using likelihood ratio.

To verify the stability of the model, several sensitivity analyses were carried out. First, considering the effects of renal function impairment and gout history on UA level, we excluded the subjects with eGFR < 45 mL/(min·1.73m^2^) or who had a history of gout for sensitivity analyses; Second, we excluded events occurring in the first 2 years of follow-up to minimize potential reverse causation; Third, to verify the robustness of the results, we excluded participants who had not completed 4 examinations or had any missing UA data for sensitivity analyses. Furthermore, Fine-Gray competing risk regression was performed to address the potentially confounding issue of competing risk, which treated deaths as competing risk events.

All analyses were performed using SAS 9.4 (SAS Institute, Cary, North Carolina). Statistical significance was set as a two-sided *P* value < 0.05.

## Results

### Baseline characteristics

We categorized the study population into three observed discrete trajectories of UA based on UA values and changing patterns during the exposure period (Fig. 2): low-stable (*n* = 34,922, 52.0%, mean UA ranged from 237.9 in 2006 to 252.6 μmol/L in 2012), moderate-stable (*n* = 26,679, 39.8%, mean UA ranged from 316.4 in 2006 to 350.56 μmol/L in 2012), and high-stable (*n* = 5494, 8.2%, mean UA from 410.9 in 2006 to 456.3 μmol/L in 2012). Baseline characteristics according to UA trajectories are presented in Table 1. The mean age was 53.12 ± 11.59 years and 50,917 (75.89%) were male. Compared with those in the low-stable, participants with a moderate-stable and high-stable trajectories of UA were more likely to be men, to be more current alcohol consumers, to be more current smokers, and to have a higher BMI, SBP, DBP, FBG, TG, CRP, UA at the first visit and baseline, while they had higher prevalence of hypertension, and taking antihypertensive drugs, and lipid-lowering drugs was more prevalent (all *P* < 0.05).

**Table 1:** Baseline characteristics of the study population according to UA trajectories

Characteristics	Total	UA trajectory group			*P*-value
		**Low-stable**	**Moderate-stable**	**High-stable**	
No. of participants	67,095	34,922	26,679	5,494	
Age (y)	53.12 ± 11.59	53.02 ± 11.04	53.48 ± 12.03	51.94 ± 12.69	< 0.001
Male (%)	50,917 (75.89)	22,171 (63.49)	23,439 (87.86)	5307 (96.60)	< 0.001
Current alcohol consumption (%)	20,564 (30.65)	7712 (22.08)	10184(38.17)	2668(48.56)	< 0.001
Current smoking (%)	22,257 (33.17)	9126 (26.13)	10,520 (39.43)	2611 (47.52)	< 0.001
Physical activity (%)	7326 (10.92)	3608 (10.33)	3086 (11.57)	632 (11.50)	< 0.001
BMI (kg/m^2^)	25.02 ± 3.23	24.45 ± 3.14	25.46 ± 3.18	26.43 ± 3.25	< 0.001
SBP (mmHg)	129.88 ± 18.59	127.89 ± 18.60	131.66 ± 18.40	133.87 ± 17.94	< 0.001
DBP (mmHg)	83.51 ± 10.36	82.07 ± 10.17	84.70 ± 10.24	86.91 ± 10.60	< 0.001
FBG (mmol/L)	5.69 ± 1.55	5.63 ± 1.65	5.75 ± 1.45	5.76 ± 1.34	< 0.001
TG (mmol/L)	1.26 (0.90, 1.93)	1.14 (0.83, 1.64)	1.40 (0.96, 2.12)	1.81 (1.19, 2.86)	< 0.001
hs-CRP (mg/L)	1.10 (0.45, 2.13)	0.84 (0.22, 1.77)	1.30 (0.68, 2.43)	1.60 (0.85, 3.10)	< 0.001
UA at the first visit (umol/L)	283.40 ± 80.08	234.94 ± 53.40	319.33 ± 57.81	416.91 ± 70.28	< 0.001
UA at baseline (umol/L)	309.44 ± 88.97	255.81 ± 61.13	350.48 ± 64.42	451.10 ± 78.98	< 0.001
Average UA (umol/L)	292.47 ± 70.14	239.84 ± 33.54	331.52 ± 32.47	437.35 ± 42.52	< 0.001
eGFR (mL/min/1.73m^2^)	91.21 ± 19.86	89.42 ± 19.55	92.91 ± 19.81	94.33 ± 20.97	< 0.001
Hypertension (%)	28,183 (42.00)	12,558 (35.96)	12,523 (46.94)	3102 (56.46)	< 0.001
Diabetes (%)	7439 (11.09)	3805 (10.90)	3021 (11.32)	613 (11.16)	0.24
Use of antihypertensive drugs (%)	14,949 (22.28)	5893 (16.87)	7061(26.47)	1995 (36.31)	< 0.001
Use of hypoglycemic drugs (%)	3991 (5.95)	2239 (6.41)	1496 (5.61)	256 (4.66)	0.001
Use of lipid-lowering drugs (%)	1481 (2.21)	564 (1.62)	717 (2.69)	200 (3.64)	< 0.001

Data are presented as mean ± SD, median (interquartile range), or n (%)

*Abbreviations*: *BMI* body mass index, *DBP* diastolic blood pressure, *eGFR* estimated glomerular filtration rate, *FBG* fasting blood glucose, *hs-CRP* high-sensitivity C reactive protein, *SBP* systolic blood pressure, *SD* standard deviation, *TG* triglycerides, *UA* uric acid

### Association between UA trajectories and outcomes

Over a median follow-up of 6.19 (4.77 ~ 6.86) years, we identified 1405 (2.09%) incident cardiac conduction block, including 478 (0.71%) incident AVB and 942 (1.40%) incident BBB. The incidence of cardiac conduction block in the total population was 3.63 per 1000 person-years. The incidence rate of cardiac conduction block was increased from 2.22 per 1000 person-year in the low-stable trajectory to 6.04 per 1000 person-year in the high-stable trajectory, and the differences among the UA trajectories were significant (log-rank test, *P* < 0.001, respectively; Table 2).

**Table 2:** Adjusted HRs and 95% CIs for risks of different events per trajectory of UA trajectories

Group	Cases/total	Incidence rate^a^	Model 1	Model 2	Model 3	Model 4	Model 5
**Cardiac conduction block**	1405/67,095	3.63					
Low-stable	549/34,922	2.22	Ref	Ref	Ref	Ref	Ref
Moderate-stable	662/26,679	4.32	1.30 (1.16–1.46)	1.31 (1.16–1.48)	1.30 (1.16–1.47)	1.36 (1.18–1.56)	1.20 (1.05–1.38)
High-stable	194/5494	6.04	1.82 (1.54–2.16)	1.88 (1.57–2.24)	1.86 (1.56–2.22)	2.04 (1.61–2.59)	1.57 (1.25–1.98)
* P*_trend_			< 0.001	< 0.001	< 0.001	< 0.001	< 0.001
**Atrioventricular block**	478/67,095	1.23					
Low-stable	170/34,922	0.84	Ref	Ref	Ref	Ref	Ref
Moderate-stable	216/26,679	1.40	1.38 (1.12–1.70)	1.41 (1.14–1.74)	1.39 (1.12–1.72)	1.54 (1.20–1.96)	1.27 (0.99–1.62)
High-stable	92/5494	2.86	2.81 (2.15–3.65)	2.97 (2.25–3.92)	2.90 (2.19–3.83)	3.62 (2.46–5.32)	2.38 (1.63–3.49)
* P*_trend_			< 0.001	< 0.001	< 0.001	< 0.001	0.003
**Bundle branch block**	942/67,095	2.43					
Low-stable	382/34,922	1.89	Ref	Ref	Ref	Ref	Ref
Moderate-stable	455/26,679	2.96	1.28 (1.11–1.47)	1.27 (1.11–1.47)	1.27 (1.10–1.47)	1.28 (1.08–1.51)	1.18 (1.00–1.40)
High-stable	105/5494	3.27	1.42 (1.14–1.77)	1.43 (1.14–1.80)	1.43 (1.13–1.79)	1.45 (1.07–1.95)	1.21 (0.90–1.63)
* P*_trend_			< 0.001	< 0.001	< 0.001	0.004	0.09

Model 1: Adjusted for age and sex

Model 2: Adjusted for variables in Model 1 plus smoking, drinking, physical activity, BMI, eGFR, hs-CRP, TG, hypertension (yes or no) and diabetes (yes or no)

Model 3: Adjusted for variables in Model 2 plus antihypertensive drugs use (yes or no), hypoglycemic drugs use (yes or no) and lipid-lowering drugs use (yes or no)

Model 4: Adjusted for variables in Model 3 plus UA at the first visit

Model 5: Adjusted for variables in Model 3 plus UA at baseline

*Abbreviations*: *BMI* body mass index, *CIs* confidence intervals, *eGFR* estimated glomerular filtration rate, *HRs* hazard ratio, *hs-CRP* high-sensitivity C reactive protein, *TG* triglycerides

^a^Case per 1000 person-years

The association between UA trajectories and the risk of different events is shown in Table 2. Compared to those in the low-stable trajectory, the adjusted HRs (95% CIs) of cardiac conduction block in the moderate-stable and high-stable trajectory were 1.30 (1.16–1.47) and 1.86 (1.56–2.22), the adjusted HRs (95% CIs) of AVB were 1.39 (1.12–1.72) and 2.90 (2.19–3.83), and the adjusted HRs (95% CIs) of BBB were 1.27 (1.10–1.47) and 1.43 (1.13–1.79), after being adjusted for variables in model 3. After additional adjustment for UA at the first visit or baseline, the association between moderate-stable and high-stable trajectory and the risk of cardiac conduction block and AVB development remained robust, while BBB risk associated with the high-stable trajectory was attenuated after additional adjustment for UA at baseline.

### Results of stratified analyses and sensitivity analyses

**Fig. 3:**
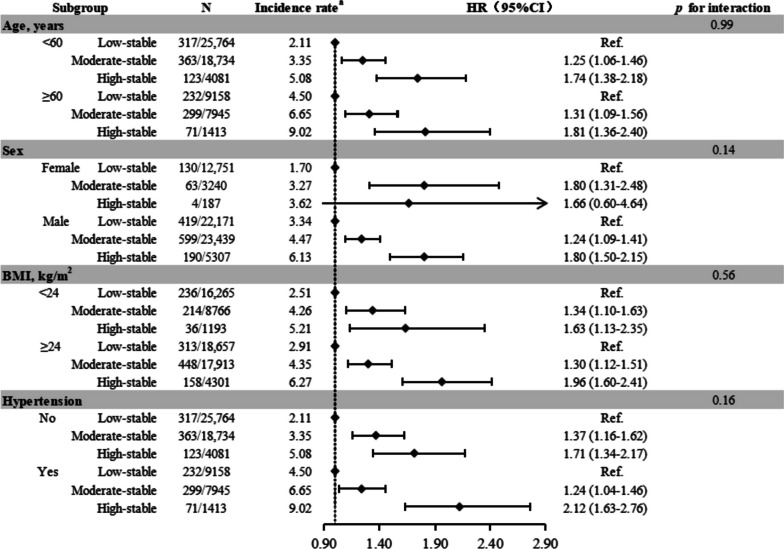
Subgroup analyses for the association with uric acid trajectories with risk of different events. ^a^ Case per 1000 person-years. HRs were adjusted for age, sex, smoking, drinking, physical activity, BMI, eGFR, hs-CRP, TG, hypertension (yes or no), diabetes (yes or no), antihypertensive drugs use (yes or no), hypoglycemic drugs use (yes or no) and lipid-lowering drugs use (yes or no). Abbreviations: BMI, body mass index; CIs, confidence intervals; eGFR, estimated glomerular filtration rate; HRs, hazard ratio; hs-CRP, high-sensitivity C reactive protein; TG, triglycerides

In the subgroup analyses, the association between UA trajectories with risk of cardiac conduction block was consistent after stratification by age (< 60 vs ≥ 60 years), sex, BMI (< 24 or ≥ 24 kg/m^2^), and history of hypertension (no or yes) (*P* for interaction > 0.05 for all, Fig. 3). Notably, the results of sensitivity analyses were consistent generally with the main analyses (Table 3). In this study, a total of 2458 (3.7%) deaths were observed. In the Fine-Gray model, after controlling for the competitive risk of death, the results were consistent with the main analyses (Table S2).

**Table 3:** Sensitive analyses of the association between UA trajectories and the risk of different events

Group	UA trajectories, HR(95%CI)		
	**Low-stable**	**Moderate-stable**	**High-stable**
**Excluding participants with follow-up < 2 years (*n* = 64,296)**			
Cardiac conduction block	Ref	1.30 (1.15–1.46)	1.86 (1.56–2.22)
Atrioventricular block	Ref	1.40 (1.13–1.73)	2.96 (2.23–3.92)
Bundle branch block	Ref	1.26 (1.09–1.46)	1.41 (1.12–1.78)
**Excluding participants eGFR < 45 (mL/min/1.73m**^**2**^**) (*n* = 65,950)**			
Cardiac conduction block	Ref	1.30 (1.15–1.46)	1.84 (1.54–2.19)
Atrioventricular block	Ref	1.38 (1.11–1.70)	2.79 (2.10–3.70)
Bundle branch block	Ref	1.27 (1.10–1.47)	1.43 (1.14–1.81)
**Excluding participants with a history of gout (*n* = 66,565)**			
Cardiac conduction block	Ref	1.30 (1.16–1.47)	1.82 (1.52–2.18)
Atrioventricular block	Ref	1.39 (1.12–1.72)	2.87 (2.16–3.81)
Bundle branch block	Ref	1.27 (1.10–1.47)	1.37 (1.08–1.73)
**Excluding participants with UA data less than 4 times (*n* = 38,854)**			
Cardiac conduction block	Ref	1.30 (1.11–1.52)	1.71 (1.36–2.16)
Atrioventricular block	Ref	1.29 (0.98–1.71)	2.58 (1.80–3.70)
Bundle branch block	Ref	1.30 (1.07–1.57)	1.31 (0.97–1.78)

HRs were adjusted for age, sex, smoking, drinking, physical activity, BMI, eGFR, hs-CRP, TG, hypertension (yes or no), diabetes (yes or no), antihypertensive drugs use (yes or no), hypoglycemic drugs use (yes or no) and lipid-lowering drugs use (yes or no)

*Abbreviations*: *BMI* body mass index, *CIs* confidence intervals, *eGFR* estimated glomerular filtration rate, *HRs* hazard ratio, *hs-CRP* high-sensitivity C reactive protein, *TG* triglycerides

## Discussion

In this prospective cohort study, three heterogeneous UA trajectories were identified, in which participants shared a similar pattern of change in UA levels over a 6-year exposure period. The main findings of this study indicate that the moderate-stable and high-stable trajectory of UA is associated with cardiac conduction block, independent of baseline UA levels. Notably, although the average UA level in the moderate-stable UA trajectory group is within the normal range, the risk of cardiac conduction block has increased. In addition, we also demonstrated that the association of distinct trajectories of UA on different outcome events had slight differences, with a higher risk of AVB than BBB.

To our knowledge, this is the first prospective cohort study to investigate the association between distinct trajectories of UA and the risk of cardiac conduction block. Over a follow-up of 388,228 person-years, we demonstrated that the high-stable UA trajectory was associated with an up to 1.86-fold increase in the risk of cardiac conduction block compared with the low-stable trajectory, and the risk remained significant even after adjusting for UA at the first visit or baseline. The only previous cross-sectional study based on one measure of baseline UA indicated that, compared with the first tertile group in patients with type 2 diabetes, the OR of cardiac conduction defects in the third tertile group of UA was 1.84 (1.20–2.90) [11]. It is worth noting that the level of UA might fluctuate substantially and be affected by lifestyle factors, environment, and diet, and therefore a single measurement of UA may be unable to examine the longitudinal association between long-term UA and cardiac conduction block. Although UA has long been shown to be antioxidant, its chronic elevation has been regarded as detrimental [20]. Previous studies indicated that higher UA trajectories were associated with altered risk of MI and all-cause mortality [21], whereas another had demonstrated that high-increasing serum urate trajectory during young adulthood was associated with incident CVD by middle age [13]. Our research is consistent with previous research findings. In the present study, by group-based trajectory modeling, we show that the high-stable UA trajectory is a risk factor for cardiac conduction block, while the increased risk is independent of traditional risk factors and baseline UA level, expanding the knowledge field of the association between UA and the risk of cardiac conduction block.

We not only indicated that the high-stable UA trajectory was associated with cardiac conduction block, but also we showed that the risk had increased in the moderate-stable UA trajectory compared with the low-stable group. The average UA level in the moderate-stable group was only 331.52 umol/L, which was far below the cut point defined for hyperuricemia (HUA). HUA was defined as > 6.8 mg/dL (408 μmol/L) according to the recommendations from the American College of Rheumatology guidelines for the management of gout 2020 [22]. According to the Chinese Expert Consensus on Hyperuricemia and Gout Treatment, the cut point defined for HUA was defined as UA levels > 420 μmol/L(7.0 mg/dL) for men, and > 360 μmol/L(6.0 mg/dL) for women [23]. The association between UA and cardiovascular disease is observed not only with frank hyperuricemia but also with UA levels considered to be in the normal to high range (> 5.2 to 5.5 mg/dL [310 to 330 μmol/L]) [24]. For instance, in the Apolipoprotein Mortality Risk study, it was demonstrated that the UA level over 281 μmol/L in males and 208 μmol/L in females was associated with an increased risk of ischemic stroke, even adjusting for potential confounders [25]. Based on the aforementioned data, we suggest that the cut-off point for hyperuricemia may be too high for CVD and cardiac conduction block. Further studies are needed to explore the optimal level of UA.

In addition, we also demonstrated that the effects of distinct trajectories of UA on different outcome events had slight differences. Compared with BBB, the high-stable trajectory of UA increased more significantly the risk of AVB. Only a few studies have focused on the differential association of UA on different sites of conduction block. In a cross-sectional study of a population with type 2 diabetes, UA level was independently associated with the risk of AVB, but not with BBB [11]. Our study showed that the high-stable trajectory of UA had a greater risk impact on AVB, while the association with BBB was attenuated after additional adjustment for UA at baseline. The different underlying risk factors for different conduction block sites might be the reason for site differences. The main risk factors for AVB include dysfunction of glycolipid metabolism, inflammation, electrolyte disturbances, and sympathetic–parasympathetic imbalance caused by autonomic neuropathy [26], while the main risk factors for BBB include hypertension, increased ventricular pressure load, and ventricular remodeling [27]. Undoubtedly, further studies are needed to investigate the mechanistic links and associations.

Subgroup analyses showed that the association between distinct UA trajectories and the risk of cardiac conduction block was not moderated by age (< 60 vs ≥ 60 years), sex, overweight, and hypertension. Notably, the distribution of UA levels varies by sex, and HUA appears to be more common in men. A prevalence of 24.4% in men and 3.6% in women was reported in 2018–2019 in China [28]. Attributed to the effects of estrogen [29], or their lifestyles, UA levels of premenopausal women tend to have lower UA levels than men [30]. Previous studies have demonstrated a stronger association between UA and the risk of cardiovascular mortality in women [31], whereas some others have demonstrated a significant association between UA and the risk of stroke was only observed in men [32], as well as no significant sex difference in the risk of MI or all-cause mortality [33]. However, our subgroup analyses showed that there was no significant interaction between sex and distinct UA trajectories in relation to the risk of conduction block disease, indicating that higher UA trajectories have similar adverse effects on the development of conduction block disease in both sexes. Besides, previous studies have shown that older age, a larger BMI, and hypertension were associated with incident conduction disease [27, 34]. Our subgroup analyses showed that when stratified by age (< 60 vs ≥ 60 years), overweight, and hypertension, the association between UA trajectories with risk of cardiac conduction block was consistent.

The precise underlying pathophysiological mechanisms of increased risk of cardiac conduction block associated with cumulative uric acid exposure remain unclear, although some hypotheses have been proposed [35–39]. First, hyperuricemia can induce primary rat cardiomyocyte apoptosis and fibrosis in vitro [35]. High levels of UA may lead to myocardial fibrosis by promoting myocardial cell hypertrophy and oxidative stress [36, 37]. Second, UA may cause electrical conduction disorders by depositing urate deposits in the conduction system [38]. Third, hyperuricemia can promote the increase of CRP [39]. The latter may promote inflammation and myocardial fibrosis through TLR4/NF-κB/TGF-β pathway [40], and arrhythmias by directly affecting calcium homeostasis in cardiomyocytes [41]. Importantly, high UA is correlated with almost all known cardiac conduction block risk factors, such as obesity, diabetes, hypertension, and coronary artery disease.

### Strengths and limitations

The strengths of the present study include large sample size, repeated measurements of UA levels, and the application of trajectory models. However, this study also has several limitations. First, we have no information about the use of UA-lowering drugs such as allopurinol and febuxostat, but we excluded the subjects with a history of gout for sensitivity analysis to ensure the reliability of the results. Second, sex distribution was unbalanced due to the nature of the study. However, subgroup analyses stratified by sex were performed, and the results showed that there was not a significant interaction between sex and UA trajectories in relation to the risk of cardiac conduction block. Third, given the observational nature of our study, there is a possibility that immortal time bias may influence the results. Therefore, caution should be exercised in extrapolating these findings. Fourth, considering the relatively brief follow-up period, further studies are warranted to substantiate and expand upon these findings in the future. Finally, given that this was an observational study, the causal relationship between UA trajectories and the risks of cardiac conduction block cannot be established.

## Conclusion

The moderate-stable and high-stable trajectories are associated with increased risk for new-onset cardiac conduction block. Monitoring UA trajectories may assist in identifying subpopulations at higher risk for cardiac conduction block.

### Supplementary Information

**Supplementary Material 1.**

## Data Availability

The datasets used and/or analyzed during the current study are available from the corresponding author on reasonable request.

## Abbreviations

AVB: Atrioventricular block
BBB: Bundle branch blocks
BMI: Body mass index
CI: Confidence interval
DBP: Diastolic blood pressure
FBG: Fasting blood glucose
Hs-CRP: High sensitive C-reactive protein
HR: Hazard ratio
MI: Myocardial infarction
OR: Odd ratio
SBP: Systolic blood pressure
TC: Total cholesterol
TG: Triglyceride
UA: Uric acid
