# Supplementary material for: Distinct uric acid trajectories are associated with incident cardiac conduction block
Source: Arthritis Res Ther. 2024 Feb 27;26:59. doi: 10.1186/s13075-024-03288-8 (PMC10898057; doi:10.1186/s13075-024-03288-8)
Supplement: Supplementary file 1 — Supplementary Material 1. [file 13075_2024_3288_MOESM1_ESM.docx]

**Supplementary Material**

Table S1. Definitions of the different conduction diseases.

Table S2. Fine-Gray model.

Figure S1. Study design and flowchart.

**Table S1. Definitions of the different conduction diseases**

| **Conduction Disease Diagnosis** | **Minnesota code** | **Definition** |
| --- | --- | --- |
| **Atrioventricular block** |  |  |
| First-degree AVB | 6-3 | P waves associated with 1:1 atrioventricular conduction and a PR interval >200 ms (this is more accurately defined as atrioventricular delay because no P waves are blocked) |
| Second-degree AVB | 6-2 | P waves with a constant rate (<100 bpm) where atrioventricular conduction is present but not 1:1 |
|  |  | Mobitz type I: P waves with a constant rate (<100 bpm) with a periodic single non-conducted P wave associated with P waves before and after the non-conducted P wave with inconstant PR intervals |
|  |  | Mobitz type II is characterized by fixed PR intervals before and after blocked beats and is usually  associated with a wide QRS complex |
| Third-degree AVB | 6-1 | No evidence of atrioventricular conduction |
| **Bundle branch block（BBB）** |  |  |
| Right BBB |  |  |
| Complete RBBB | 7-2 | 1. QRS duration of 120 ms or longer in the presence of normal sinus rhythm or supraventricular rhythm;  2.R or rSR′complex in lead V1;  3.rS in leads V5, V6, I, or aVL with prolonged shallow S wave. |
| Incomplete RBBB | 7-3 | Same QRS morphology criteria as complete RBBB but with a QRS duration between 110 and 119 ms |
| Left bundle branch block |  |  |
| Complete LBBB | 7-1 | 1. QRS duration of 120 ms or longer in the presence of normal sinus rhythm or supraventricular rhythm (not atrial fibrillation);  2. QS or rS complex in lead V1;  3. broad R waves in leads I, aVL, V5-V6 (or an rS pattern in V5-V6);  4. absence of Q waves in leads V5, V6, or I. |
| Incomplete LBBB | 7-6 | 1. QRS duration between 110 and 119 ms in adults；  2. Presence of left ventricular hypertrophy pattern；  3. R peak time >60 ms in leads V4, V5, and V6；  4. Absence of Q wave in leads I, V5, and V6 |
| Left anterior fascicular block |  | 1. QRS duration <120 ms；  2. Frontal plane axis between −45° and −90°；  3. R-peak time in lead aVL of ≥45 ms；  4. qR (small r, tall R) pattern in lead aVL；  5.rS pattern (small r, deep S) in leads II, III, and aVF |
| Left posterior fascicular block |  | 1. QRS duration <120 ms；  2. Frontal plane axis between 90° and 180°；  3.rS (small r, deep S) pattern in leads I and aVL；  4. qR (small q, tall R) pattern in leads III and aVF |
| Nonspecific intraventricular conduction delay | 7-4 | QRS duration >110 ms where morphology criteria for RBBB or LBBB are not present |

**Table S2. Fine-Gray model**

| **group** | **Model 1** | **Model 2** | **Model 3** | **Model 4** | **Model 5** |
| --- | --- | --- | --- | --- | --- |
| **Cardiac conduction block** |  |  |  |  |  |
| Low-stable | Ref. | Ref. | Ref. | Ref. | Ref. |
| Moderate-stable | 1.31 (1.16-1.47) | 1.32 (1.17-1.49) | 1.31 (1.16-1.48) | 1.38 (1.19-1.59) | 1.22 (1.06-1.41) |
| High-stable | 1.82 (1.54-2.15) | 1.87 (1.57-2.23) | 1.85 (1.55-2.21) | 2.07 (1.63-2.62) | 1.59 (1.25-2.01) |
| **Atrioventricular block** |  |  |  |  |  |
| Low-stable | Ref. | Ref. | Ref. | Ref. | Ref. |
| Moderate-stable | 1.39 (1.13-1.71) | 1.42 (1.14-1.75) | 1.40 (1.13-1.73) | 1.55 (1.21-2.00) | 1.29 (1.01-1.64) |
| High-stable | 2.79 (2.15-3.64) | 2.96 (2.23-3.92) | 2.89 (2.17-3.84) | 3.65 (2.49-5.35) | 2.41 (1.67-3.50) |
| **Bundle branch block** |  |  |  |  |  |
| Low-stable | Ref. | Ref. | Ref. | Ref. | Ref. |
| Moderate-stable | 1.28 (1.11-1.48) | 1.28 (1.11-1.49) | 1.28 (1.11-1.48) | 1.30 (1.09-1.54) | 1.19 (1.00-1.43) |
| High-stable | 1.41 (1.13-1.76) | 1.43 (1.14-1.80) | 1.42 (1.13-1.79) | 1.46 (1.08-1.98) | 1.22 (0.90-1.67) |

Model 1: Adjusted for age and sex;

Model 2: Adjusted for variables in Model 1 plus smoking, drinking, physical activity, BMI, eGFR, hs-CRP, TG, hypertension (yes or no) and diabetes (yes or no)；

Model 3: Adjusted for variables in Model 2 plus antihypertensive drugs use (yes or no), hypoglycemic drugs use (yes or no) and lipid-lowering drugs use (yes or no);

Model 4: Adjusted for variables in Model 3 plus UA at the first visit；

Model 5: Adjusted for variables in Model 3 plus UA at baseline.

Abbreviations: BMI, body mass index; CIs, confidence intervals; eGFR, estimated glomerular filtration rate; HRs, hazard ratio; hs-CRP, high-sensitivity C reactive protein; TG, triglycerides.

Excluded participants without data on electrocardiogram during follow-up (n = 9586)

Excluded participants with uric acid data of less than 3

times or without data of electrocardiogram at baseline

(n = 2939)

**Figure S1. Flow chart of study participants**

Participants for this study (n = 67,095)

Individuals who participated at least three examinations during 2006-2012 (n=87,669)

Excluded participants with a history of cardiac

conduction block, myocardial infarction, heart failure,

or atrial fibrillation at baseline (n =6891)

Excluded participants using beta-blocker or non-dihydropyridine calcium-channel blockers at baseline (n =1158)
